# Microstructural Characterization of AlCrCuFeMnNi Complex Concentrated Alloy Prepared by Pressureless Sintering

**DOI:** 10.3390/ma17102378

**Published:** 2024-05-15

**Authors:** Tiago Silva, Augusto Lopes

**Affiliations:** Department of Materials and Ceramic Engineering, CICECO-Aveiro Institute of Materials, University of Aveiro, 3810-193 Aveiro, Portugal

**Keywords:** complex concentrated alloys, microstructure, powder metallurgy, pressureless sintering

## Abstract

A significant and increasing number of studies have been dedicated to complex concentrated alloys (CCAs) due to the improved properties that these metallic materials can exhibit. However, while most of these studies employ melting techniques, only a few explore powder metallurgy and pressureless sintering as production methods. In this work, a microstructural characterization of AlCrCuFeMnNi CCA samples obtained by powder metallurgy and pressureless sintering using mixtures of powders with different compositions was carried out. One batch of samples (B1) was prepared using commercial powders of Al, Cr, Cu, Fe, Mn, and Ni. Another batch (B2) used mixtures of CrFeMn, AlNi, and Cu powders. A third set of samples (B3) was obtained by adding 1% at. of Mg to the B2 powder. The samples were characterized by X-ray diffraction, scanning and transmission electron microscopy, energy dispersive spectroscopy, density measurements, and hardness tests. Thermodynamic calculations were also used to complement the microstructural characterization. All the obtained samples exhibited high relative density and hardness values. However, B3 samples showed a higher hardness, attributed to the finer distribution of oxide particles, which was promoted by the presence of Mg during sintering. These last samples presented a hardness/density ratio of 62 HV/(g cm^−3^), surpassing that of some martensitic stainless steels and nickel–titanium alloys.

## 1. Introduction

Complex concentrated alloys (CCAs) or multi-principal element alloys (MPEAs) have attracted much interest over the past two decades [1,2,3]. The distinguishing feature of these alloys is that they contain several major elements, unlike conventional alloys, introducing new possibilities for developing materials with improved properties such as higher hardness, lower thermal conductivity, high strength at room and high temperatures, improved wear behaviour, and better corrosion resistance [1,2,3,4].

The term CCA is a general definition that includes metallic alloys containing three or more components with atomic concentrations between 5% and 35% [2,3,5]. Although this definition also includes high entropy alloys, it does not specifically consider the configurational entropy value or the presence of a single solid solution phase [2]. CCAs may have various phases, such as solid solutions, intermetallics, or a mixture of both [2]. The SS phases in these alloys are usually classified by their atomic packing sequence as face-centred cubic (FCC), body-centred cubic (BCC), and hexagonal close-packed (HCP) [2,6,7].

Most of the CCAs reported in the literature are constituted by three or more principal elements, such as Al, Co, Cr, Cu, Fe, Ni, Ti, V, or Mn, and sometimes refractory elements like W or Mo. Elements such as Cu, Ni, Mn, and Co tend to promote the formation of FCC phases [5], while Al, Cr, W, V, and Ti facilitate the formation of BCC phases [5]. HCP phases are less common and usually include rare earth elements such as Sc, Y, and lanthanides [7]. In recent years, an increasing number of studies have employed thermodynamic calculations to predict the phase evolution in multicomponent systems [2,8].

One of the most studied CCAs is CoCrFeMnNi [9], which exhibits at room temperature values of tensile strength and elongation that can reach 496 MPa and 61.7%, respectively [10]. With the addition of aluminium, the tensile strength of this CCA increases up to 529 MPa and the elongation decreases to 47.2% [10]. Despite some of these alloys showing mechanical properties comparable to Ni-based superalloys, the use of cobalt can restrict some engineering applications due to the high cost and toxicity of this element. Co-free alloys were investigated by Chen et al. [11], replacing Co with Mn in AlCrCuFeCoNi alloys. They found that the obtained CCA is composed by FCC and BCC phases and that the addition of Mn favours the formation of the BCC phase, leading to an improvement of hardness and a decrease in ductility. Other studies on the AlCrCuFeMnNi system showed some interesting properties, such as hardness around 500 HV for as-cast samples, resistance to annealing softening, relatively low cost, and good oxidation resistance at room and high temperatures [12,13]. They also reported that Al plays an important role in this system. The increasing of Al content reduces ductility and increases hardness, showing the best combination of mechanical properties at concentrations around x = 0.25 [14].

The properties of CCAs are determined not only by their chemical composition but also by the processing conditions. Most CCAs studies involving Al, Cr, Cu, Fe, Mn, and Ni were carried out with the arc melting technique [10,11,15,16] and few studies use powder metallurgy techniques. This latter approach offers several advantages, such as the good homogeneity of the obtained products, an ability to be used in systems with elements with a broad range of melting points, and being easily adapted for large-scale production [17,18]. The powder metallurgy method is a process that usually includes a mechanical alloying or milling step and shaping through compaction and sintering to consolidate the powder [2,19]. The sintering process of CCA powders is often performed by non-conventional methods. Spark plasma sintering (SPS) is one of the most common techniques reported in the literature for sintering these materials [1,20]. However, this technique is limited to samples with small sizes and simple shapes [18]. Although it can be used to consolidate parts with different shapes and dimensions and requires relatively simple equipment, pressureless sintering presents some challenges such as the presence of oxides on the surface of the particles that hinder the densification process, limiting the properties of the obtained products [18,21]. In the case of Al, several studies [22,23,24,25,26,27] have shown that the presence of oxide layers on the particles limits the diffusion process and wettability due to the liquid phase formed during the sintering. These effects can be mitigated by decreasing the oxides’ amount using powders with a lower oxygen content, control of the processing conditions, such as the sintering atmosphere, and the addition of Mg. This element tends to oxidize more than Al, forming MgO and Al_2_MgO_4_ which promotes the rupture of the oxide layer, enhancing the diffusion and the densification of the samples [26].

The main objective of this work was to perform a microstructural characterization of AlCrCuFeMnNi CCA samples obtained by powder metallurgy and pressureless sintering using powders with different compositions and to study the effect of Mg addition on the microstructure and hardness of the sintered samples.

## 2. Materials and Methods

In this work, three different batches of CCA samples were produced by powder metallurgy from mixtures containing equimolar amounts of Al, Cr, Cu, Fe, Mn, and Ni. The first batch of samples (B1) was prepared from commercial powders of the elements with purity higher than 99.5% and particle sizes between 12 µm and 250 µm (Al, Cr, Fe, Mn, and Ni below to 45 µm and Cu around 250 µm, all supplied by Alfa Aesar, Ward Hill, MA, USA) in equimolar proportions (16.7% mol of each element). A second set of samples (B2) with the same nominal chemical composition was produced from mixtures of powders of AlNi, CrFeMn, and Cu. The AlNi powder was supplied by Alfa Aesar with purity higher than 99.5% and grain size around 60 µm. The CrFeMn alloy was produced by melting Cr, Fe, and Mn powders at 1350 °C for 30 min in a reducing atmosphere of 90% argon and 10% hydrogen to decrease the oxygen content in the melt. After melting, the slag was removed and the material was crushed to a size less than 350 µm. The Cu powder was subjected to a heat treatment at 1000 °C during 60 min using the same atmosphere. These treatments were carried out for reducing the oxygen content of the raw materials. This last methodology was also used to produce a third set of samples (B3) through the addition to a B2 initial mixture of 1% at. Mg powder with purity higher than 99.5% and particle size less than 45 µm.

The powders were wet milled for 15 h in an atmosphere of 90% helium and 10% hydrogen using a planetary mill equipment (Retsch PM100, Haan, Germany) operating at 250 rpm with a ball/powder ratio of 10:1. Steel vials and balls were used as the milling media and toluene as a process controlling agent. After vacuum drying at room temperature, the obtained powders were cold uniaxially pressed with 750 MPa to produce cylindrical samples with approximately 10 mm diameter and 4 mm height. Apparent density value, using weigh and geometric measurements, was evaluated for each sample before sintering at 1050 °C during 60 min in a tubular furnace using a 90% helium and 10% hydrogen atmosphere.

The powders and the sintered samples were characterized by X-ray diffraction (XRD) using a Panalytical X’pert Pro diffractometer (Malvern, UK) operating with Cu-Kα radiation, scanning electron microscopy (SEM, Hitachi SU-70, Tokyo, Japan), transmission electron microscopy (TEM, JEOL 2200FS, Tokyo, Japan), and energy dispersive X-ray spectrometry (EDS) using a Bruker Quantax (Billerica, MA, USA) and an Oxford Ultim Max (Oxfordshire, UK) system (in SEM and TEM, respectively). From sintered samples, polished and fractured surfaces were analysed by SEM. TEM samples were first mechanically ground to a thickness of 20 µm and ion beam polished until a hole was formed (Gatan 691 Precision ion polishing system, Pleasanton, CA, USA) using 1.5 kV to 4 kV and angles between 4° and 2°. Particle size distributions of the powder mixtures after milling were analysed by laser scattering (Horiba LA-960, Kyoto, Japan). For density measurement, the Archimedes principle and Vickers hardness test (2 kgf load, Wilson VH1102, Lake Bluff, IL, USA) were used to characterize the sintered samples.

The relative density of the samples was evaluated using the equation:

Relative density = D_exp_/D_th_ × 100 (1)

where D_exp_ is the measured density value and D_th_ is the theoretical density calculated from the relative amount of each phase in the sample and its tabulated density obtained by XRD [28].

The analysis of the microstructural evolution of the samples during sintering was complemented by thermodynamic calculations of the equilibrium phases using FactSage 7.3 software combined with the SGTE 2017 thermodynamic database [29].

## 3. Results and Discussion

### 3.1. Powder Characterization

**Figure 1:**
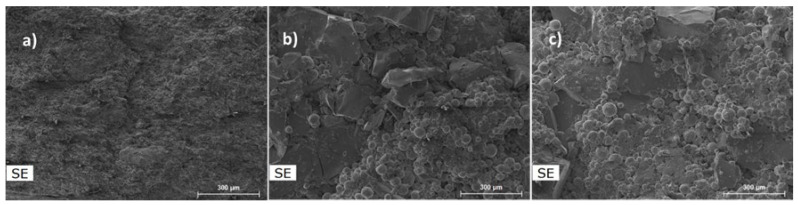
SEM images of (**a**) B1, (**b**) B2, and (**c**) B3 powder before milling.

Figure 1 presents SEM images of B1, B2, and B3 powders before the milling process. Powders B2 and B3 present a mixture of irregular particles and almost spherical particles with larger average sizes than B1 powder. In the large irregular particles, the presence of Cr, Fe, and Mn in similar atomic proportions was detected by EDS analysis (corresponding to the CrFeMn powder), while in the rounded particles was detected Cu (corresponding to the Cu powder) or Al and Ni in similar atomic proportions (corresponding to AlNi powders). In B3 powder, the analyses also confirmed the presence of Mg particles with an almost spherical shape. In all samples, the measured average composition was very close to the nominal one.

**Figure 2:**
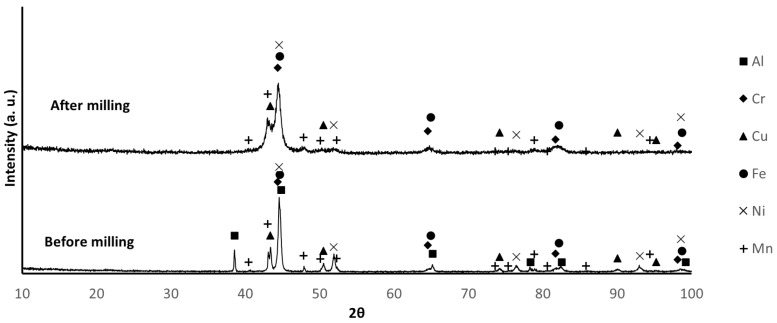
XRD patterns of B1 powder before and after milling. (PDF card index: Al (04-012-3402); Cr: (04-003-2918); Cu: (01-070-3039); Fe: (04-002-8852); Ni: (04-004-6807); Mn (04-007-1944) [28]).

**Figure 3:**
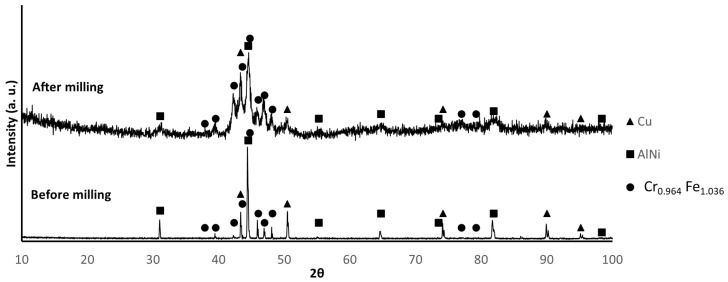
XRD patterns of B2 powder before and after milling. (PDF card index: Cu: (01-070-3039); AlNi (04-012-6340); Cr_0.964_Fe_1.036_ (01-071-7533) [28]).

**Figure 4:**
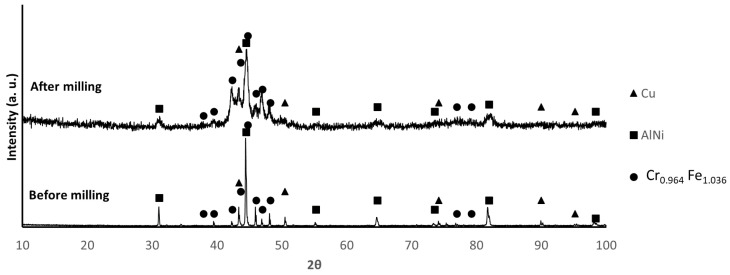
XRD patterns of B3 powder before and after milling. (PDF card index: Cu: (01-070-3039); AlNi (04-012-6340); Cr_0.964_Fe_1.036_ (01-071-7533) [28]).

Figure 2, Figure 3 and Figure 4 show the X-ray diffractograms of B1, B2, and B3 mixtures before and after milling. The identified phases are consistent with the presence of Al, Cr, Cu, Fe, Mn, and Ni in B1 samples, as well as Cu, AlNi, and FeCrMn in B2 and B3 samples. It is worth mentioning that, because the diffraction data for FeCrMn were not available, the identification of the diffraction maxima of this phase was based on experimental diffractograms of the alloy prepared by melting, which was almost coincident with the diffraction pattern tabulated for the Fe_1.036_ Cr_0.964_ phase. The main difference between the diffractograms before and after milling is the broadening of the diffraction peaks, attributable to the decrease in crystallite size and the increase in lattice strain due to milling.

**Figure 5:**
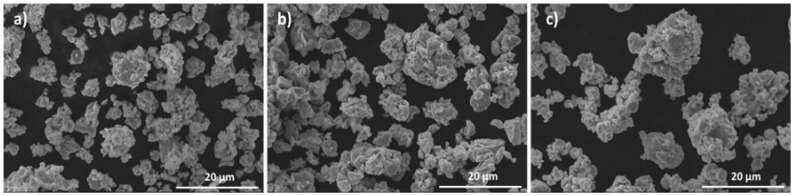
SEM images of (**a**) B1, (**b**) B2, and (**c**) B3 powder after milling.

**Figure 6:**
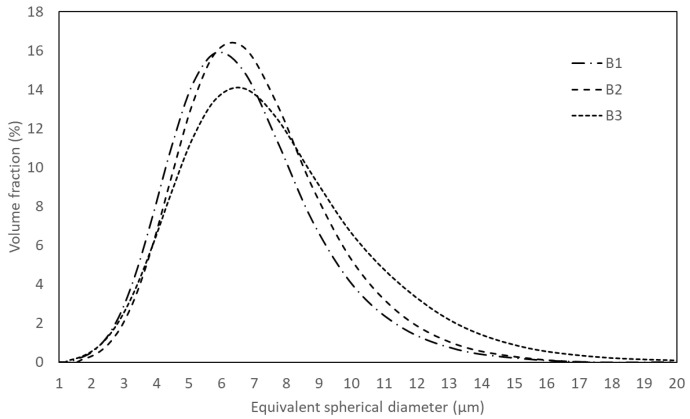
Particle size distribution of the powders after milling.

**Figure 7:**
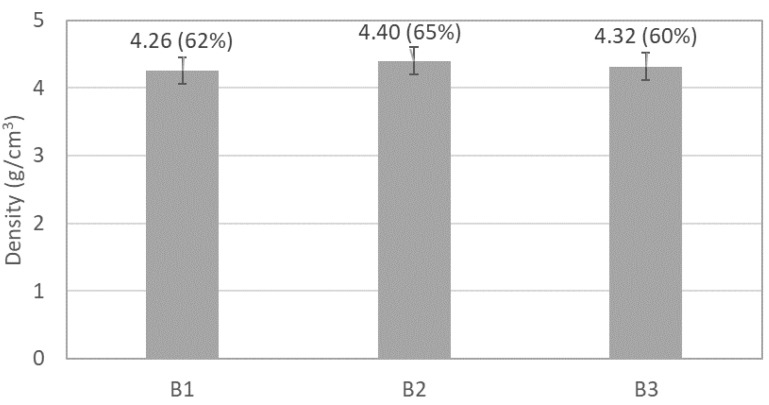
Density of the samples after uniaxial pressing. The relative density values are presented in brackets.

The SEM (Figure 5) and EDS analysis showed that the mixtures after milling are mainly composed of agglomerates of particles with a maximum size of around 3 µm and a high level of chemical homogeneity. The degree of agglomeration is similar for the three batches of powders, as shown by the particle size distribution curves obtained by laser scattering technique (Figure 6). These results also indicate that the milled powders have a unimodal distribution with an average size of around 6 µm and a maximum size of less than 20 µm. Although the powder B3 exhibits a slight increase in the fraction of particles with a larger size, the differences between the powders did not influence the degree of compaction by uniaxial pressing, and all samples presented a similar density value before sintering (between 4.26 g/cm^3^ and 4.40 g/cm^3^, which corresponds to 62% and 65% of relative density, respectively), as shown in Figure 7.

### 3.2. Sample Characterization after Sintering

**Figure 8:**
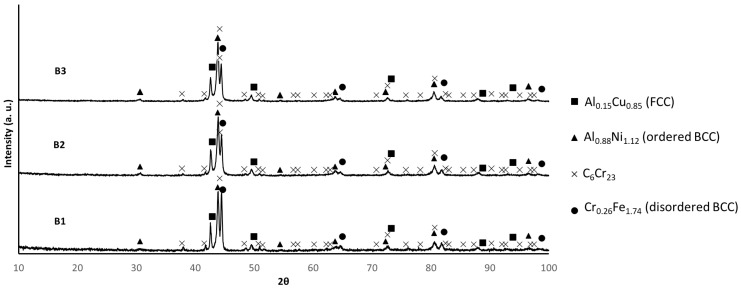
XRD patterns of B1, B2, and B3 sintered samples. (PDF card index: Al_0.15_Cu_0.85_ (01-077-6740); Al_0.88_Ni_1.12_ (04-002-1233); Cr_23_C_6_ (04-004-3124); Cr_0.26_Fe_1.74_ (00-034-0396) [28]).

Figure 8 shows the XRD results of the sintered samples. The patterns are similar for all samples, presenting diffraction maxima compatible with the presence of C_6_Cr_23_, Al_0.88_Ni_1.12_ (ordered BCC), Cr_0.26_Fe_1.74_ (disordered BCC), and Al_0.15_Cu_0.85_ (FCC) [2,11].

**Figure 9:**
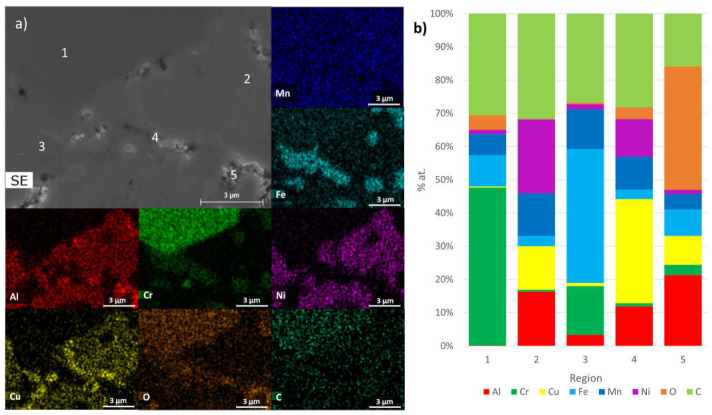
(**a**) SEM image and distribution maps of Al, Cr, Cu, Fe, Mn, Ni, O, and C; (**b**) results of quantitative analyses by EDS on B1 sintered sample at the points identified in SEM image.

**Figure 10:**
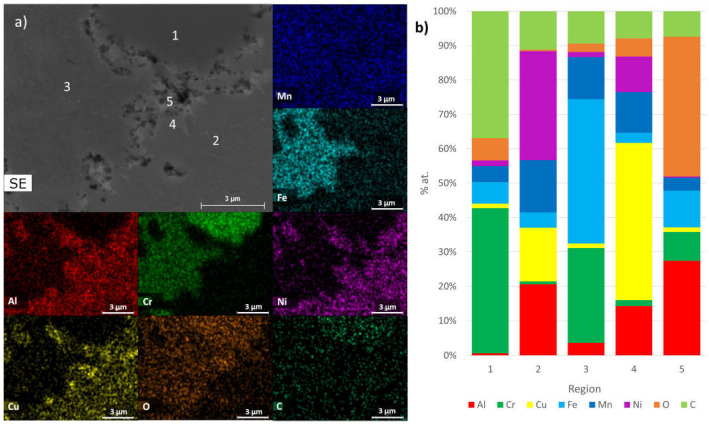
(**a**) SEM image and distribution maps of Al, Cr, Cu, Fe, Mn, Ni, O, and C; (**b**) results of quantitative analyses by EDS on B2 sintered sample at the points identified in SEM image.

**Figure 11:**
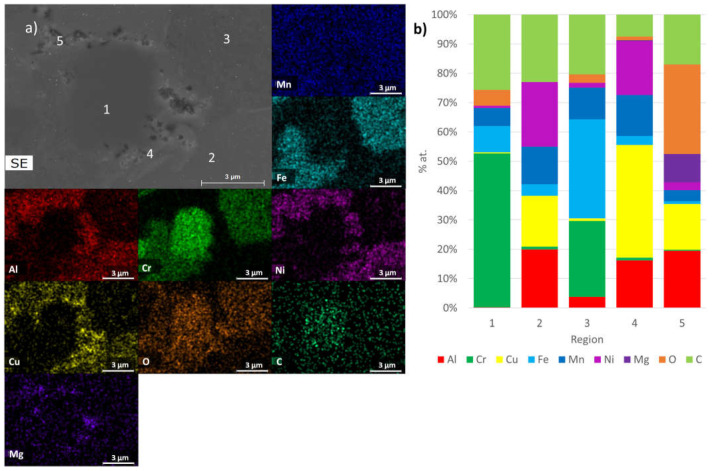
(**a**) SEM image and distribution maps of Al, Cr, Cu, Fe, Mn, Ni, O, C, and Mg; (**b**) results of quantitative analyses by EDS on B3 sintered sample at the points identified in SEM image.

These results are in good agreement with the chemical composition determined through EDS analysis. As depicted in Figure 9, Figure 10 and Figure 11, SEM-EDS analyses of polished surfaces enabled the identification of areas rich in C-Cr (point 1 in B1, B2, and B3), Al-Ni (point 2 in B1, B2, and B3 samples), Cr-Fe (point 3 in B1, B2, and B3 samples), and Al-Cu (point 4 in B1, B2, and B3 samples), which is consistent with the presence of Cr_23_C_6_, Al_0.88_Ni_1.12_ (ordered BCC), Cr_0.26_Fe_1.74_ (disordered BCC), and Al_0.15_Cu_0.85_ (FCC), respectively. These analyses also revealed the presence of oxygen, either coming from the initial powders or introduced during processing. The high concentrations of Al and O (point 5 in B1 and B2 samples) and Al, Mg, and O (point 5 in B3 sample) are consistent with the presence Al_2_O_3_ and Al_2_MgO_4_, respectively.

**Figure 12:**
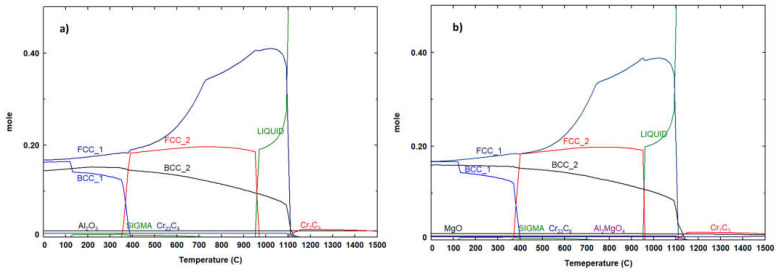
Relative amount of the phases predicted by the thermodynamic calculations in equilibrium at different temperatures for (**a**) B1 and B2 and (**b**) B3 samples.

Figure 12 presents the results of thermodynamic calculations for a mixture with equimolar concentrations of Al, Cr, Cu, Fe, Mn, and Ni (B1 and B2 samples) and 1% atomic Mg (B3 samples). In these calculations, the presence of 5% at. C and 2% at. O (average amounts measured by SEM-EDS on B2 samples) was also considered. As indicated in Table 1, there was a good agreement between the predicted phases in thermodynamic equilibrium at room temperature and the experimental XRD and EDS results. Both compositions exhibited similar phases, with the exception of MgO and Al_2_MgO_4_, which were predicted only for B3 samples. MgO, however, was not experimentally detected, possibly due to the partial volatilization of Mg as a result of it high partial vapor pressure at temperatures used for sintering [30]. The sigma (tetragonal) phase, rich in Cr, Mn, and Fe, was not experimentally detected, likely due to its small amount and similar composition to the Cr_0.26_Fe_1.74_ phase.

It should be noted that a Laves phase AB2, usually observed in compositions containing Mg, Cu, and Ni (mostly as MgCu_2_ and MgNi_2_), was not detected in B3 samples. This phase has high hardness but reduces the toughness of the sample and is often undesirable [31]. This suggests that the Mg added to B3 samples primarily contributed to the formation of magnesium oxide and aluminium–magnesium oxide.

**Figure 13:**
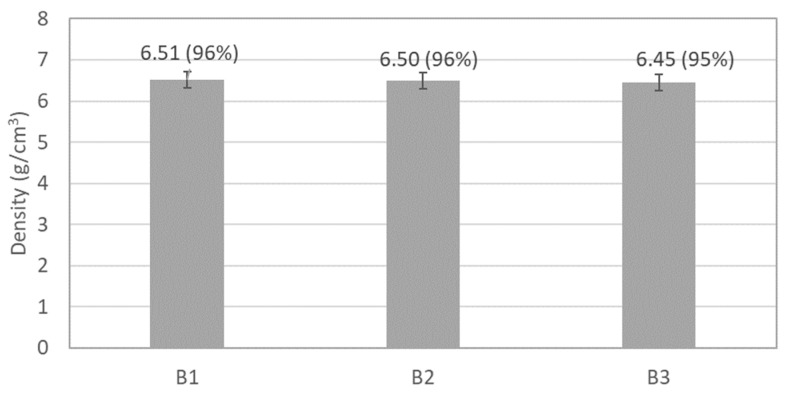
Density of the sintered samples. The relative density values are presented in brackets.

Another significant result from the thermodynamic calculations in Figure 12 is the formation of a liquid phase after around 950 °C. It is well-known that the presence of a wetting liquid phase during sintering facilitates particle rearrangement and enhances mass transfer rates between them [32], resulting in reduced residual porosity, as observed in Figure 9, Figure 10 and Figure 11. The similar amount of liquid phase predicted for both compositions agrees with the minor differences in density values (averages between 6.45 g/cm^3^ and 6.51 g/cm^3^, which correspond to 95% and 96% of relative density if the phase proportions predicted by the thermodynamic calculations are considered) exhibited by the samples after sintering (Figure 13).

**Figure 14:**
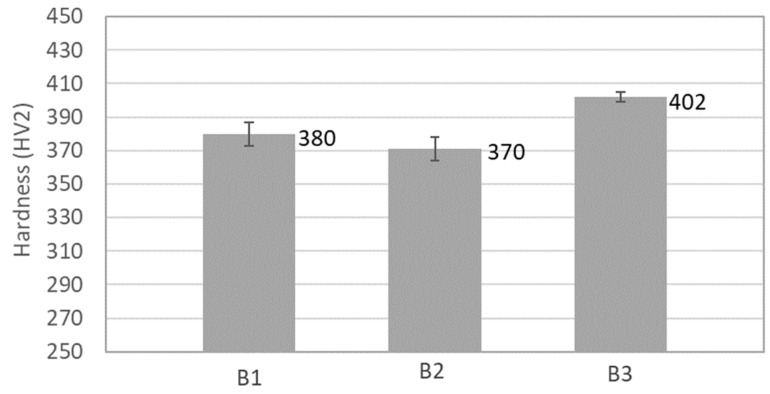
Vickers hardness values of the sintered samples.

In previous studies conducted on samples produced by casting with a composition similar to that studied in this work [12,13,14,15], the presence of an ordered BCC phase rich in Al and Ni and a disordered BCC phase containing mainly Fe and Cr was observed [12,13]. Additionally, the presence of a Cu-rich FCC phase was reported in some of these studies [13,14]. These phases significantly influence the properties of the CCA [2], particularly its hardness, toughness, yield stress, and tensile strength. While samples with higher amounts of BCC phases tend to exhibit higher hardness and strength but lower toughness, FCC phases usually promote higher toughness but lower hardness and strength [2]. Considering the results of XRD, EDS, and thermodynamic calculations, all sintered samples exhibited higher amounts of BCC phase (considering both the ordered and disordered phases) than FCC phase, which is consistent with the relatively high hardness values (ranging between 370 HV and 402 HV) of the samples (Figure 14). However, despite no differences in the relative amounts of these phases being detected, B3 samples displayed higher hardness than B1 and B2 samples, achieving a hardness-to-density ratio of 62 HV/(g cm^−3^), surpassing that of some martensitic stainless steels and nickel–titanium alloys.

**Figure 15:**
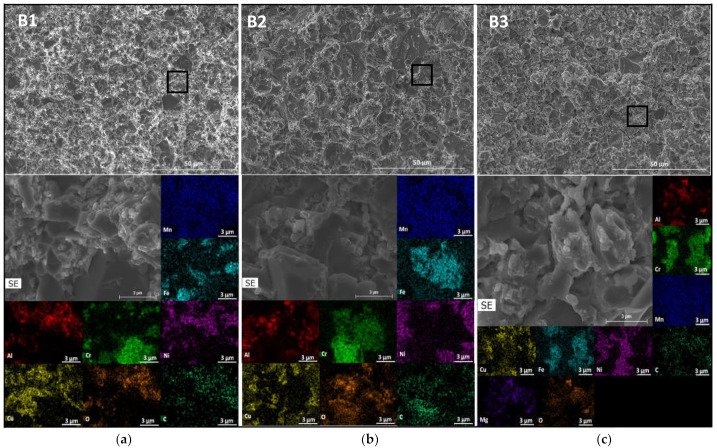
SEM images and EDS of Al, Cr, Cu, Fe, Mn, Ni, O, and C distribution maps on (**a**) B1, (**b**) B2, and (**c**) B3 sintered samples. The squares in the top images correspond to the areas where EDS analyses were carried out.

Since no differences were detected by SEM or TEM analyses in the size or distribution of metallic phases and residual porosity that could justify the changes in the hardness value, the observed increase in this property for the B3 samples should be attributed to the addition of Mg to the initial powder. A more detailed analysis of fracture surfaces (Figure 15) confirmed the presence of oxides in all samples, with sample B1 having the highest amount. This indicates that the treatment carried out on the initial materials used to produce the B2 and B3 samples (CrFeMn alloy obtained by melting, thermal treatment of Cu, and commercial AlNi powders) resulted in a decrease in the oxide content. However, this decrease did not affect the hardness of the B2 sample but led to an increase in the B3 sample (whose only difference was the presence of Mg).

**Figure 16:**
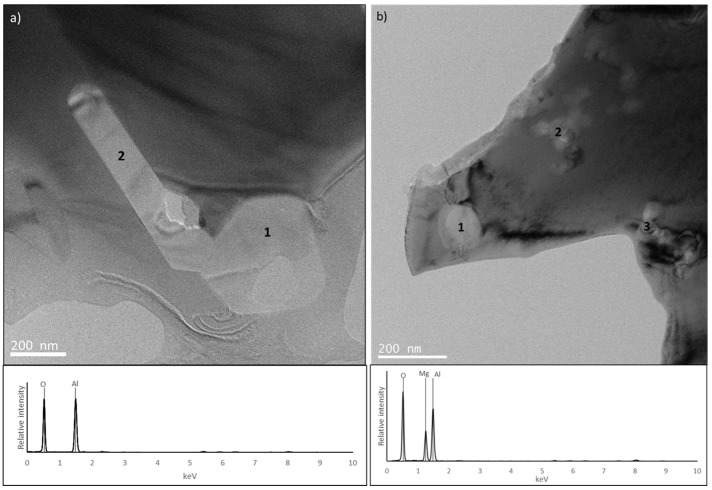
TEM image of (**a**) B2 and (**b**) B3 sample. For each image is also presented an EDS spectrum from the identified regions.

Since the initial amounts of oxygen in samples B2 and B3 were similar, it was expected that after sintering, the oxide content would also be similar in these samples, which would not therefore justify the differences in the hardness values. However, the B2 and B3 samples showed differences in the size and distribution of the oxides (Figure 15 and Figure 16). In the former samples, the oxides were present in the form of aluminium oxide particles with sizes around 0.5 µm, mainly concentrated along the grain boundaries (these features were also observed in B1 samples). In contrast, in B3 samples, the oxides were primarily in the form of more uniformly distributed particles of magnesium oxide and aluminium–magnesium oxide with a size of a few tens of nanometres. Therefore, the higher hardness value presented by B3 samples can be attributed to a strengthening effect resulting from the smaller size and more uniform distribution of the oxide particles promoted by the presence of Mg in the initial powder. Indeed, the enhancement of strength by a distribution of nanometric hard particles has been observed in many metallic materials (see for example [33]), and Mg is often used as a sintering additive for Al and its alloys to break down the oxide layers on the surface of the particles [25,26].

**Figure 17:**
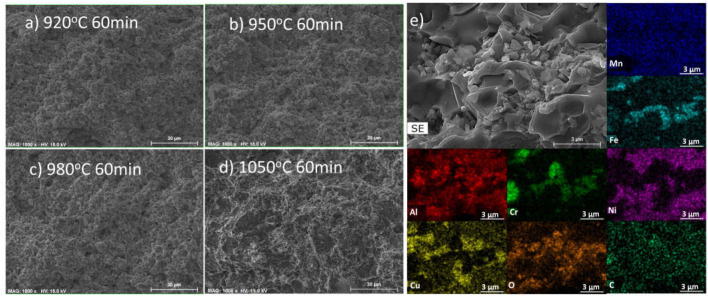
SEM image of B2 samples after sintering at (**a**) 920 °C, (**b**) 950 °C, (**c**) 980 °C, and (**d**) 1050 °C for 60 min; (**e**) magnified image and EDS distribution maps of Al, Cr, Cu, Fe, Mn, Ni, O, and C on the sample sintered at 1050 °C.

To analyse the formation of the coarser and more concentrated oxide particles when Mg is absent, additional microstructural observations were carried out on B2 samples sintered at temperatures between 920 °C and 1050 °C. The results obtained (Figure 17) showed that the samples sintered at 920 °C and 950 °C exhibited high homogeneity, with no visible areas with a high concentration of oxides. In contrast, in samples sintered at 980 °C and 1050 °C, oxide particles were observed at the grain boundaries, with their presence being more evident in the sample sintered at the higher temperature. Considering that the thermodynamic calculations predict the formation of a liquid phase around 950 °C, these results suggest that the coalescence and heterogeneous distribution of the oxides are promoted by the presence of this liquid phase through a mechanism that involves its segregation and preferential concentration at the grain boundaries.

## 4. Conclusions

AlCrCuFeMnNi CCA samples with around 95% of relative density were successfully produced in this work through powder metallurgy and pressureless sintering, using mixtures of powders with different compositions. The following results were found:

- All samples exhibited high hardness (with average values ranging between 371 HV and 402 HV), attributed to the significant amount of BCC phases detected in the samples after sintering. The samples with higher hardness values (B3) were achieved through the addition of 1% at. Mg to the initial powder mixture. These samples presented a hardness/density ratio of 62 HV/(g cm^−3^), surpassing that of some martensitic stainless steels and nickel–titanium alloys;
- A good agreement was observed between the phases identified by XRD and EDS in the sintered samples and those predicted by thermodynamic calculations. These calculations also predicted that a liquid phase was formed during sintering;
- The presence of oxides in relatively small amounts was detected in all samples. In the samples without Mg (B1 and B2 samples), these oxides were present as aluminium oxides particles with a size of around 0.5 µm, mainly concentrated along the grain boundaries. By contrast, in the samples with Mg (B3 samples), the oxides were present mostly in the form of more uniformly distributed particles of magnesium oxide and aluminium–magnesium oxide with a few tens of nanometres in size. These changes, attributed to the presence of Mg during sintering, can explain the higher hardness value exhibited by the B3 samples.

## Figures and Tables

**Table 1:** Phases identified by XRD and EDS and predicted by thermodynamic calculations for 25 °C.

Phases Predicted by the Thermodynamic Calculations (at 25 °C)	Phase Identified by XRD	EDS Analysis
B1, B2 and B3 FCC_1 rich in Cu and Al	B1, B2 and B3 Al_0.15_Cu_0.85_ (FCC)	B1, B2 and B3 Region 4 of Figure 9, Figure 10 and Figure 11
B1, B2 and B3 BCC_1 rich in Fe, Mn, Al and Cr	B1, B2 and B3 Cr_0.26_Fe_1.74_ (disordered BCC)	B1, B2 and B3 Region 3 of Figure 9, Figure 10 and Figure 11
B1, B2 and B3 BCC_2 rich in AlNi	B1, B2 and B3 Al_0.88_Ni_1.12_ (ordered BCC)	B1, B2 and B3 Region 2 of Figure 9, Figure 10 and Figure 11
B1, B2 and B3 C_6_Cr_23_	B1, B2 and B3 C_6_Cr_23_	B1, B2 and B3 Region 1 of Figure 9, Figure 10 and Figure 11
B1, B2 and B3 Sigma	B1, B2 and B3 Not detected	Not detected
B1 and B2 Al_2_O_3_	B1, B2 and B3 Not detected	B1 and B2 Region 5 of Figure 9 and Figure 10
B3 MgO	B1, B2 and B3 Not detected	Not detected
B3 Al_2_MgO_4_	B1, B2 and B3 Not detected	B3 Region 5 of Figure 11

## Data Availability

The raw data supporting the conclusions of this article will be made available by the authors on request.
